# Insights into genetic and clinical profiles of triple A syndrome in Sudanese children

**DOI:** 10.3389/fendo.2025.1617552

**Published:** 2025-08-22

**Authors:** Salwa A. Musa, Mohamed A. Abdullah, Samar S. Hassan, Eliane Streiff, Franziska Lange, Omer O. Babiker, Areej A. Ibrahim, Hiba A. Elshafie, Angela Huebner, Katrin Koehler, Friederike Quitter

**Affiliations:** ^1^ Department of Pediatrics and Child Health, Faculty of Medicine, Al-Neelain University, Khartoum, Sudan; ^2^ Department of Pediatric Endocrinology and Diabetes, Gaafar Ibn Auf Pediatric Tertiary Hospital, Khartoum, Sudan; ^3^ Department of Pediatrics and Child Health, Faculty of Medicine, University of Khartoum, Khartoum, Sudan; ^4^ Division of Pediatric Endocrinology and Diabetes, Department of Pediatrics, Faculty of Medicine and University Hospital Carl Gustav Carus, Technische Universität Dresden, Dresden, Germany; ^5^ Department of Pediatrics, Faculty of Medicine and Health Sciences, Omdurman Islamic University, Khartoum, Sudan; ^6^ School of Medicine, Ahfad University for Women, Khartoum, Sudan

**Keywords:** triple A syndrome, Allgrove syndrome, alacrima, achalasia, primary adrenal insufficiency, familial cases, Sub-Saharan Africa

## Abstract

**Introduction:**

Triple A syndrome (OMIM*231550) is a rare autosomal recessive disorder characterized by achalasia, alacrima, adrenal insufficiency, and neurological features. It is caused by functional impairment of the nucleoporin ALADIN due to mutations in the *AAAS* gene. Limited data exists on triple A syndrome from Sub-Saharan African and Arab countries. Our objective is to perform a comprehensive clinical and genetic study in Sudanese patients diagnosed with triple A syndrome.

**Methods:**

The clinical diagnoses were based on characteristic clinical and laboratory findings. Genetic testing was conducted in 20 families, encompassing 31 patients, revealing six different *AAAS* mutations.

**Results:**

A previously described mutation in exon 9 (c.934C>T) was present in 35%, and the known Arabic founder mutation c.1331+1G>A (intron 14) was found in 30% of the families. In addition, two novel mutations, including an 8 bp-deletion at the exon 4/intron 4 junction (c.394_399+2delCTGTCTGT) and a 1 bp-deletion in exon 9 (c.852delG) were identified.

**Discussion:**

Genotype-phenotype analyses highlighted significant variability in symptom occurrence, age of onset, and disease severity. Consistent with the high consanguinity rates in Sudan, most mutations (95%) occurred in a homozygous state. In conclusion, triple A syndrome is likely underdiagnosed in Sudan and exhibits significant variability in phenotypic presentation even among affected individuals within the same family or mutation.

## 1 Introduction

Triple A syndrome, also known as Allgrove syndrome (OMIM#231550), is an autosomal recessive multisystemic disorder. It is a very rare disease with an extremely low prevalence of fewer than 1 in 1 000 000 (www.orpha.net). The condition is initially characterized by the clinical triad of alacrima, achalasia, and adrenal insufficiency (1). Over time, two-thirds of the patients present with progressive neurological impairment affecting the central, peripheral, and autonomic nervous systems, prompting some authors to suggest the term 4A syndrome (2, 3). Additional symptoms include a dysmorphic facial appearance, xerostomia, dental caries, palmar and plantar hyperkeratosis, gait disturbances, microcephaly, developmental delays, and delayed puberty (4, 5). Patients with triple A syndrome exhibit significant variability in clinical presentation, including the age of symptom onset, the frequency and severity, and the discordance between phenotypes and genotypes. This variability is evident even among affected members from the same family or mutation, further contributing to the heterogeneity of this syndrome. Alacrima is the earliest and most consistent symptom, occurring in over 90% of patients, whereas achalasia is present in 75-85% of the patients and primary adrenal insufficiency (PAI) in about 85% (3, 6).

Triple A syndrome is caused by mutations in the *AAAS* gene on chromosome 12q13, which consists of 16 exons encoding a 546 amino acid protein termed ALADIN (Alacrima–Achalasia–Adrenal Insufficiency Neurologic disorder). ALADIN is a protein of the nuclear pore complex that controls the transport of macromolecules between the nucleus and the cytoplasm. Most mutations in *AAAS* result in a delocalization of ALADIN to the cytoplasm (7), which affects the nucleocytoplasmic transport of specific proteins, including DNA repair proteins (8). This renders cells more vulnerable to oxidative stress, leading to selective tissue degeneration (9–11). The precise role of the ALADIN protein remains largely unknown, but studies investigating the pathogenic mechanisms of various mutations have shown an impaired nuclear import of DNA repair proteins and increased oxidative stress within the cell. ALADIN I482S fibroblasts were found to be more vulnerable to oxidative stress, which combined with impaired import of DNA repair proteins, led to increased cell death. This may contribute to the progressive nature of the disease (9).

The *AAAS* gene is expressed in a wide range of human tissues, leading to a variety of symptoms in the affected patients. Notably, there is a particularly high expression in the adrenal gland, gastrointestinal tract, and brain (12, 13). To date, 91 unique mutations have been identified across the *AAAS* gene according to “The Human Gene Mutation Database” (https://www.hgmd.cf.ac.uk/ac/gene.php?gene=AAAS). The majority of the mutations are nonsense and frameshift mutations, which are likely to result in a truncated, non-functional protein, followed by missense and splice-site mutations (14). Mutations were identified across the entire *AAAS* gene, indicating the absence of specific hotspots. However, several of the most frequently observed mutations cluster in specific ethnicities. For instance, mutations c.1432C>T (p.Arg478*) and c.787T>C (p.Ser263Pro) are common in Europe, c.771delG (p.Arg258fs) is prevalent in China, c.762delC (p.Ser255fs) is found in India, while the widely reported splice-site mutation c.1331+1G>A is noted in Africa and the USA (15). The c.1331+1G>A substitution was first identified in unrelated North African patients with triple A syndrome, indicating a founder effect (12).

Many studies have been conducted worldwide; however, apart from a few studies from North Africa, reports from Sub-Saharan Africa and Arab countries are limited, primarily consisting of small studies or case reports (16, 17). Here, we report clinical and genetic characterization of 31 children from 20 Sudanese families. To our knowledge, no studies have simultaneously examined both aspects in a sizable cohort from Sub-Saharan Africa and Arab countries to date.

## 2 Materials and methods

### 2.1 Subjects and DNA extraction

The clinical and biochemical presentations of all patients with clinically suspected triple A syndrome who presented to Gaafar Ibn Auf (GIA) Children’s Tertiary Hospital in Khartoum, Sudan, between 2018 and 2023 were reviewed. The initial diagnosis of triple A syndrome was based on characteristic clinical signs and symptoms, including alacrima, achalasia, adrenal insufficiency, as well as autonomic and neurological features. Adrenal insufficiency was confirmed using the following criteria: Early morning serum cortisol levels below the reference range (< 6 μg/dL), elevated ACTH levels (> 100 pg/mL), and cortisol levels below 18 μg/dL following an ACTH stimulation test (18). Additional diagnostic support was provided by pathological findings on barium swallow studies and positive Schirmer’s test results.

**Figure 1:**
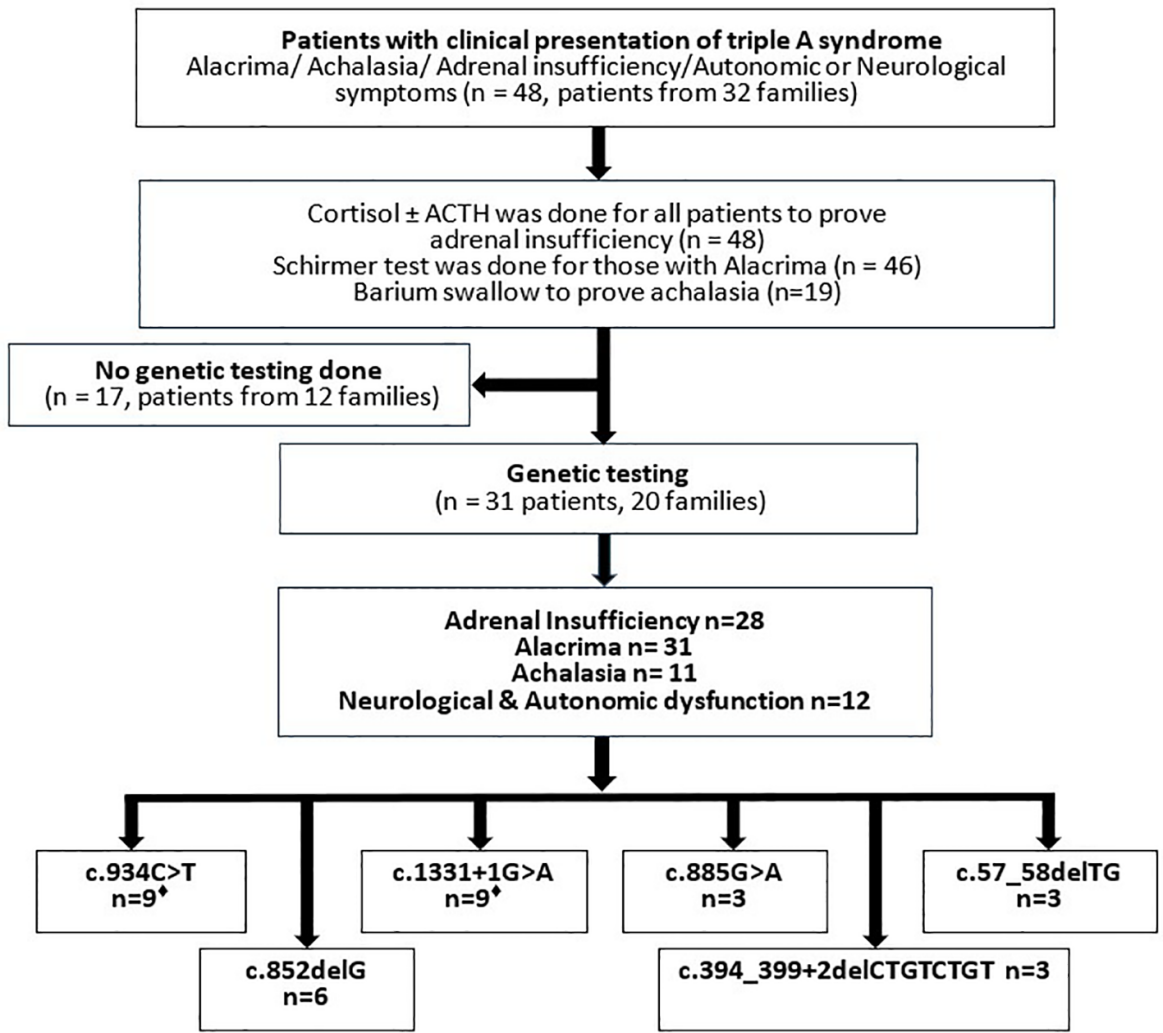
Flowchart showing the course of the investigation of Sudanese patients admitted with triple A syndrome. Laboratory and instrumentational examinations, result of genetic testing of the Sudanese cohort. ^♦^Two patients carry the mutations in a heterozygous form.

The total number of patients who fulfilled the clinical and biochemical criteria for triple A syndrome was 48 patients from 32 families. From those, 31 patients from 20 families were included in this study, as other patients were not available or had passed away before the genetic testing was performed. Clinical data for those patients included personal demographics, age at symptom onset, symptom duration, growth parameters, parental consanguinity, family history of similar conditions or sibling deaths, as well as the diagnostic workup and management provided (**Figure 1**). EDTA blood samples were collected from 31 patients from 20 families, along with their siblings and parents who consented to participate in the study. Genomic DNA was extracted from blood cells using the QIAamp DNA Mini Kit (QIAGEN GmbH, Hilden, Germany) according to standard protocols. The study adhered to the principles outlined in the Declaration of Helsinki.

### 2.2 PCR and sanger sequencing

Coding sequences of the 16 exons of *AAAS*, including exon-intron boundaries, were amplified from genomic DNA by polymerase chain reaction (PCR) using gene-specific primers (Eurofins Genomics Germany GmbH, Ebersberg, Germany) (**Table 1**) and PCR standard protocols.

**Table 1:** Primer sequences for amplification of genomic DNA of *AAAS*.

Exon	Forward primer sequence (5´-3´)	Reverse primer sequence (5´-3´)	Product size (bp)
1	GTCCTTCTTTCCCGTTAGTC	TGCCTCCTTTCCCCAGTAGTCCCCGA	368
2	GGTTGGAGATGGCACGGAAT	ACTGTGACCCAGGAAACCCTT	238
3	CAGAGGGGATCAGAGTAAGC	GGGTGAGTTCAAGAATATGGAGAG	171
4	CAGAAGGACCTGGGGAGTGCC	GAGTAGGATGGGGACACAGCAAATG	251
5	GATGACTTCAGAGGGTAGGAGTGG	CAGGAGGAATGAGAATATGGTGAGC	270
6	TTCTGGGCTAGGGTAGGGAT	CCAAGGCTGGCAAGGGAAGGTG	218
7	TAACTGCACTCTGGTCCCTG	ACCATGCCTAGCATTGCAC	271
8	TTGGGGATGGCTTCTTGTCA	ACCGAGTGAGGAACACTTTTGCCAG	237
9	CTCGTTTCTGGCTCTTTGTA	GTTCTAAAAGTTGGACCTACCTC	315
10-11	TCTGTCAAGGGAGGTAGGTC	TCTTACCAAAGGTTGCTGCC	432
12-13	TTGATGCCTTTGGTGAGCTG	GGACATGGGCAGAGGAGAAC	424
14-15	CTCAGCCCACAGCCATCTTC	AGAGCCATACAGCAGCCAAG	490
16	CTTGGCTGCTGTATGGCTCT	GCAACTCCCTGGAAAAGACA	365

PCR products were purified using AcroPrep 96-well filter plates filled with hydrated illustra™ Sephadex^®^ G-50 DNA grade F chromatography resin (Sigma-Aldrich Chemie GmbH, Taufkirchen, Germany), and sequenced on an ABI 3130XL genetic analyzer using BigDye Terminator Cycle Sequencing Kit 1.1 (Applied Biosystems Inc., Foster City, CA). Data were analyzed using Mutation Surveyor V5.1.2 software (SoftGenetics, LLC. State College, PA, USA).

### 2.3 RNA analysis

For RNA preparation, the blood of relevant patients (Patients 23, 24) and healthy controls was collected in PAXgene Blood RNA tubes (BD, Heidelberg, Germany). Total RNA was prepared using PAXgene Blood RNA Kit (QIAGEN GmbH, Hilden Germany). After reverse transcription of messenger ribonucleic acid (mRNA) with Go Script™ Reverse Transcription System (Promega GmbH, Mannheim, Germany), the sequences of exons 2 to 6 were amplified using the forward primer (exon 2) 5’-GATCCCCTAAAGACCCCTGG-3’ and the reverse primer (exon 6) 5’-CTGCTGGCATTATACACACG-3’.

## 3 Results

### 3.1 Patients

We studied 20 Sudanese families, including 31 patients (15 male, 16 female) from Gaafar Ibn Auf Pediatric Hospital, the main tertiary hospital in Khartoum, Sudan. All of the probands exhibited at least two symptoms of the diagnostic triad: achalasia, alacrima, and adrenal insufficiency. The medical diagnosis was confirmed by the detection of a mutation in the *AAAS* gene in all 31 patients. Clinical and genetic details are summarized in **Table 2**.

**Table 2:** Clinical findings and genetic analysis of 31 patients with triple A syndrome (M, male; F, female).

Case	Family	Sex	Age of presentation (years)	Alacrima/ dry eyes	Adrenal insufficiency	Achalasia/ swallowing difficulties	Neurological dysfunction	Autonomic dysfunction	Symptoms at presentation			Additional features	Mutation	
									Hyperpig-mentation	Alacrima	Adrenal crisis		DNA level	Protein level
1	F1	F	4	x	x				x	x	x	Learning difficulties	c.934C>T	p.Arg312*
2	F2	M	3	x	x				x	x	x	Growth failure, short stature	c.934C>T	p.Arg312*
3	F2	F	5	x	–				–	x	–		c.934C>T	p.Arg312*
4	F3	M	1.5	x	x			x	x	x	x		c.1331+1G>A	splice defect
5	F3	M	0.5	x	–				–	x	–		c.1331+1G>A	splice defect
6	F4	M	6	x	x	x	x	x	x	x	x	Recurrent seizure, hypertensive encephalopathy, facial dysmorphism	c.885G>A	p.Trp295*
7	F4	M	3	x	x		x	x	x	x	x	Recurrent seizure, hypertensive encephalopathy, facial dysmorphism	c.885G>A	p.Trp295*
8	F4	F	2	x	x				x	x	–	–	c.885G>A	p.Trp295*
9	F5	F	6	x	x	x			x	x	x	Learning difficulties, facial dysmorphism	c.1331+1G>A	splice defect
10	F6	M	5	x	x				x	x	x		c.934C>T	p.Arg312*
11	F7	F	6	x	x	x			x	x	x	Facial dysmorphism	c.1331+1G>A	splice defect
12	F8	F	8	x	x	x		x	x	x	x	Growth failure, recurrent abdominal pain, candidiasis, facial dysmorphism	c.852delG	p.Trp284Cysfs*7
13	F9	M	5	x	x	x	x		x	x	–	Recurrent seizure, epilepsy, facial dysmorphism	c.934C>T	p.Arg312*
14	F10	F	2	x	x				x	x	x	Poor weight gain, facial dysmorphism	c.1331+1G>A	splice defect
15	F11	F	5	x	x	x			x	x	x	Learning difficulties, short stature	c.934C>T	p.Arg312*
16	F12	M	9	x	x				x	x	–		c.852delG	p.Trp284Cysfs*7
17	F13	F	13	x	x	x			x	x	–		c.852delG	p.Trp284Cysfs*7
18	F13	F	15	x	x	x			x	x	–		c.852delG	p.Trp284Cysfs*7
19	F14	M	9	x	x	x	x	x	x	x	x	Excessive nasal discharge, learning difficulties, facial dysmorphism, psychological disturbances	c.57_58delTG	p.Tyr19*
20	F14	F	7	x	x				x	x	–		c.57_58delTG	p.Tyr19*
21	F14	M	2	x	–				–	x	–		c.57_58delTG	p.Tyr19*
22	F15	M	3.7	x	x		x		x	x	x	Facial dysmorphism	c.394_399+2delCTGTCTGT	splice defect
23	F15	M	5	x	x		x		x	x	x	Facial dysmorphism	c.394_399+2delCTGTCTGT	splice defect
24	F16	M	4	x	x		x		x	x	x	Excessive thick nasal discharge, facial dysmorphism	c.394_399+2delCTGTCTGT	splice defect
25	F17	F	4	x	x		x	x	x	x	x	Facial dysmorphism	c.934C>T	p.Arg312*
26	F18	F	5	x	x				x	x	x		c.1331+1G>A	splice defect
27	F18	M	3	x	x				x	x	–		c.1331+1G>A	splice defect
28	F19	F	2	x	x				x	x	x		c.934C>T c.1331+1G>A	p.Arg312* splice defect
29	F19	F	2	x	x				x	x	x		c.934C>T c.1331+1G>A	p.Arg312* splice defect
30	F20	M	9	x	x	x	x		x	x	x	Excessive nasal discharge, learning difficulties, facial dysmorphism	c.852delG	p.Trp284Cysfs*7
31	F20	F	11	x	x	x	x	x	x	x	x	Excessive nasal discharge, learning difficulties, facial dysmorphism, psychological disturbances	c.852delG	p.Trp284Cysfs*7

### 3.2 Clinical findings

The gender distribution in our cohort was balanced (48.4% male, 51.6% female). The median age at presentation was 5.5 years (range 6 months - 15 years). Alacrima was consistently observed in all 31 patients, while hyperpigmentation indicating the presence of ACTH-resistant PAI was present in 28/31 patients (90%) at the time of presentation. The primary presenting symptom in the index patients was adrenal insufficiency, manifesting as adrenal crises, hypoglycemic seizures, and hyperpigmentation. The other three patients presented with alacrima as the only symptom, with a family history of a confirmed affected sibling with triple A syndrome that warranted genetic testing to prove the diagnosis.

The duration of PAI symptoms before medical consultation ranged from one month to 36 months. Swallowing difficulties were elicited in 11 patients (35%), with the youngest being five years old. These patients underwent barium swallow studies to confirm the achalasia diagnosis. The triad of alacrima, achalasia, and adrenal insufficiency was detected in 11 patients (35%), while alacrima and adrenal insufficiency were detected in 28 patients (90%) at presentation. Twelve patients (39%) exhibited autonomic and neurological features, including orthostatic hypotension, increased sweating, gait abnormalities, progressive neurological dysfunction, and recurrent convulsions. Other clinical findings detected at presentation include facial dysmorphism in 14/31 patients (45%) and growth failure or short stature in 12 patients (39%). Other rare clinical aspects included two patients with hypertensive encephalopathy requiring intensive care unit admission, four patients (13%) reported unexplained excessive thick nasal mucus, psychological disturbances (seen in one patient) and learning difficulties in six patients (19%).

Consanguinity was observed in 16 families (80%). Additionally, eight families (40%) reported premature deaths of one or more siblings who had shown similar symptoms and died with symptoms suggestive of adrenal crisis. Cortisol was low (< 6 mcg/dl) in 25 out of 26 patients who had serum cortisol tested (mean = 1.7 mcg/dl), and borderline low in one patient (7.9 mcg/dl), while ACTH levels were high (> 100 pg/ml) (mean = 1535 pg/ml) in 23 patients who were tested. The patient with borderline cortisol levels had high ACTH in addition to a genetic diagnosis and an affected family member. The five patients who did not undergo cortisol testing had alacrima, a genetic diagnosis, and a family history of a confirmed triple A diagnosis. Three of those five did not have PAI symptoms, which makes future follow-up examinations necessary. The same applies to the eight patients who did not undergo ACTH testing, three of whom did not have PAI and the remaining five exhibited low cortisol levels (< 6 mcg/dl) in addition to genetic diagnosis of triple A syndrome. Additionally, Schirmer tests were positive for all patients, indicating alacrima or hypolacrima to varying degrees. Hydrocortisone treatment was initiated for 11 patients (mean dosage per day 12–15 mg), while 17 older patients were prescribed prednisolone due to the unavailability of hydrocortisone. Three patients undergo annual monitoring of their cortisol levels to evaluate for potential PAI. Three patients, unfortunately died at different ages despite receiving medical management. One male patient (F4) passed away at the age of 9 years following severe hypertensive encephalopathy that required prolonged pediatric intensive care unit admission. Patient F8, a 16-year-old female who suffered from severe achalasia and experienced multiple adrenal crises despite adequate steroid therapy, also succumbed. The third case was an 8-year-old male patient (F15), whose family had discontinued hydrocortisone treatment for some time due to unavailability, leading to a fatal adrenal crisis.

### 3.3 Sanger sequencing

Sequencing of the coding exons and adjacent intronic regions of the *AAAS* gene (OMIM *605378, Transcript-ID ENST00000209873; GenBank NM_015665) revealed six different *AAAS* mutations in this cohort, which are summarized in **Table 3**. Two of the mutations are novel *AAAS* mutations (c.394_399+2delCTGTCTGT and c.852delG) that have not been previously described in any other case of triple A syndrome nor in the variant aggregation database GnomAD 4.1 (https://gnomad.broadinstitute.org/). The four other mutations are previously recognized mutations (12, 19, 20).

**Table 3:** Spectrum of *AAAS* variants in Sudanese patients with triple A syndrome.

Exon	Mutation	Protein level	Number of families (patients)	Allele frequence in GnomAD total/African;African American	Reference
Exon 1	c.57_58delTG	p.Tyr19* (Y19X)	1 (3)	0.00003779/0.0007864	(19)
Exon4/Intron 4	c.394_399+2 delCTGTCTGT	splice defect	2 (3)	0	new
Exon 9	c.852delG	p.Trp284Cysfs*7 (W284fs)	4 (6)	0	new
Exon 9	c.885G>A	p.Trp295* (W295X)	1 (3)	0	(20)
Exon 9	c.934C>T	p.Arg312* (R312X)	7 (9^♦^)	0.000003717/0.00002665	(12)
Intron 14	c.1331+1G>A	splice defect	6 (9^♦^)	0.00004213/0.00002665	(12)

^♦^Two patients are carrying the mutations in a heterozygous form. GnomAD 4.1 (https://gnomad.broadinstitute.org/).

### 3.4 RNA analysis

**Figure 2:**
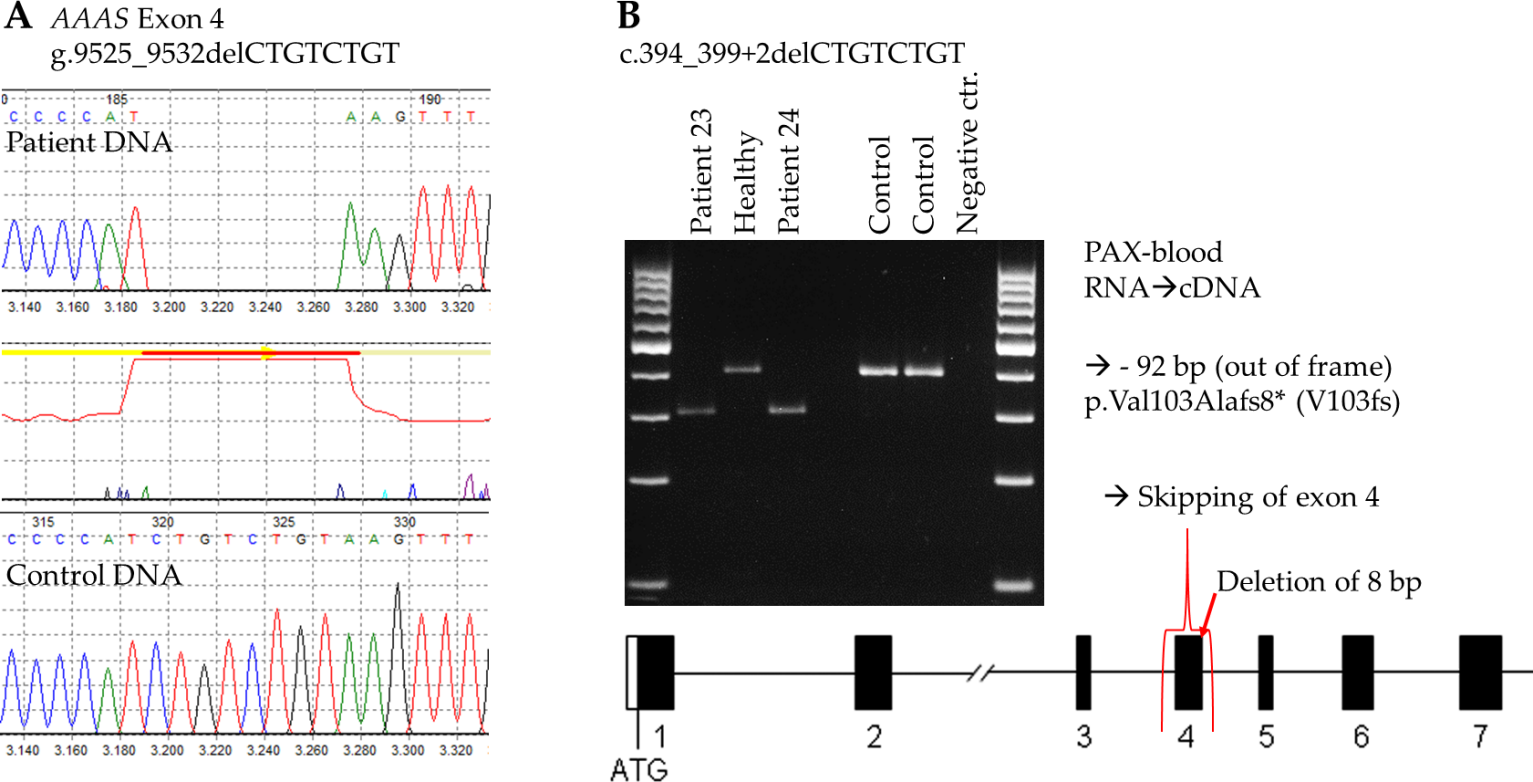
Investigation of the novel *AAAS* mutation c.394_399+2delCTGTCTGT at DNA and RNA level. **(A)** Electropherogram of WT and patient DNA sequencing showing the deletion of 8 base pairs at the border of exon 4 and intron 4. **(B)** RT-PCR of two patients showing that exon 4 is missing due to the 8 bp deletion, as compared to the RT-PCR of a healthy family member and two independent controls.

To investigate how the genetic alteration at donor splice site of exon 4 impact mRNA processing we used RNA from PAX-blood from two patients (patient 23 and 24) and a healthy control from family 15 and two independent controls. We revealed that cDNA with the mutation c.394_399+2delCTGTCTGT caused exon 4 to be skipped during RNA translation due to the splice mutation (**Figure 2**). As a result, this mutation leads to a frameshift after amino acid 103 and introduces a premature stop codon, which likely causes a nonsense-mediated RNA decay or impairs the function of the truncated ALADIN protein. We were able to confirm this splice result using the latest prediction tool SpliceAI (21).

## 4 Discussion

Sudan is an African and Arab country with multiple tribes, diverse ethnic groups, and a high rate of consanguinity, an environment that leads to a high prevalence of autosomal recessive conditions. While awareness of genetic disorders is growing, many remain underdiagnosed due to the varying nonspecific symptoms that overlap with more common childhood diseases. This is the first comprehensive report on Sudanese patients with triple A syndrome. In this study, genetic analysis of 31 patients revealed six different mutations in the *AAAS* gene associated with significant clinical variability even among family members with the same mutation(s), consistent with previous studies (4, 14, 22). The consanguinity rate in this cohort was 80%, which aligns with findings from similar studies (23). Notably, there were no sex-based differences in the presentation of triple A syndrome, with a nearly balanced gender distribution (male-to-female ratio of 0.94). The main presenting symptom was adrenal insufficiency manifested as severe adrenal crisis and hypoglycemic seizure, while alacrima was present in all patients upon evaluation. Alacrima is typically the first and most common symptom to be reported in triple A syndrome patients, although it is often overlooked, despite being present from birth (24, 25). Since isolated alacrima is rare in children, its diagnosis should prompt careful evaluation for triple A syndrome, particularly when accompanied by additional symptoms or a family history of unexplained sibling deaths (26, 27). Adrenal insufficiency is the leading cause of mortality in undiagnosed triple A syndrome patients, which is usually a consequence of ACTH insensitivity and is typically associated with hypoglycemia, chronic vomiting, hyperpigmentation, and, in some cases, sudden death due to adrenal crisis (28). This condition affected 28 (90%) of our patients and was the primary presentation leading to triple A syndrome diagnosis. In our cohort, all patients with adrenal insufficiency exhibited generalized or localized hyperpigmentation, 75% experienced severe adrenal crises, and eight families reported sibling deaths likely attributable to adrenal crisis. The age of PAI onset varied widely, even within the same affected family (F2, F3, F14, F18), and 90% percent of the children in this cohort exhibited PAI symptoms within the first decade of life, with a mean age of onset at 5.5 years. 55% developed PAI symptoms before the age of five, highlighting an early onset of the disease and the need for early recognition and management. Triple A syndrome is the second most common cause of primary adrenal insufficiency (PAI) in Sudan, possibly due to distinctive features that facilitate clinical diagnosis once adrenal insufficiency is recognized (23). Mineralocorticoid deficiency, reported in up to 15% of patients in demographically similar cohorts, is typically associated with the degeneration of the zona glomerulosa. Variability in prevalence across different studies underscores the need for further investigation into this component of the syndrome’s clinical presentation (22, 29). Achalasia was observed in 11 patients (35%) in this cohort, a lower prevalence than reported in some larger studies, potentially due to the younger age of the study participants. Two patients were diagnosed with both achalasia and adrenal insufficiency concurrently, while others developed achalasia later, consistent with findings from other studies (15, 22).

Neurological dysfunction in triple A syndrome often emerges in the 2^nd^ or the 3^rd^ decade(s) of life, indicating the neurodegenerative nature of the disease. However, early presentation in childhood was also observed in our cohort, with 80% of patients presented before the age of 10 years. This may be due to recurrent hypoglycemic seizures or other mechanisms that have yet to be fully elucidated (7, 30). Early diagnosis and treatment appeared to slow down neurological progression, as observed in some families in this study. In these cases, late presenters showed severe progressive neurological symptoms (Patients no. 4, 6, 7, 19) whereas early diagnosed siblings (Patients no. 5, 8, 20, 21) showed milder progression and preserved neurological functions. Long-term follow-up and further studies are needed to elucidate the mechanisms leading to this observation.

Hypertensive encephalopathy, linked to autonomic dysfunction, is a rare complication of triple A syndrome (31). We reported two male siblings who presented with fever, hypertensive crisis, and convulsions. One fully recovered after three weeks in intensive care, while the other progressed to a complete neurovegetative state and died at the age of nine years, highlighting the clinical variability among affected siblings.

Excessive thick nasal mucus discharge, a newly documented feature in this cohort, was observed in 13% of our patients who were associated with three different mutations. One family sought a CT scan, revealing adenoid enlargement and a complicated middle concha bullosa. The other patients had similar symptoms for years, often misattributed to chronic respiratory tract bacterial infections. This discharge may be linked to autonomic dysfunction, but the exact mechanism remains unclear and is yet to be uncovered.

In our Sudanese cohort, six different *AAAS* gene mutations were identified, four of which were previously described. In general, triple A syndrome-associated mutations can occur in all 16 exons of the *AAAS* gene in a homozygous or compound heterozygous form (5, 32, 33). In our cohort, mutations were identified in exons 1, 4, 9, and 14. These mutations included predominantly homozygous point mutations and small deletions.

The c.934C>T mutation, one of the most common in our cohort (29%), was first described in an Algerian patient by Tullio-Pelet et al. in the year 2000 (12). All nine affected patients presented with alacrima and adrenal insufficiency, except for patient 3, who had alacrima and a confirmed genetic diagnosis of triple A syndrome but has not developed adrenal insufficiency to date.

The common Arabic founder mutation c.1331+1G>A was also present in 29% of our patients. It is one of the most common *AAAS* gene mutations worldwide and is most prevalent in North Africa (17). The mutation was initially identified in multiple consanguineous North African triple A patients, and it may represent a regional founder mutation (12). This variant is a substitution at the start of intron 14 that disrupts the splice donor site leading to abnormal transcripts (34). Patients with c.1331+1G>A mutation presented alacrima and adrenal insufficiency except for patient 5, who exhibited alacrima only. Achalasia was observed in just two patients (Patients 9 and 11). Ten percent of the cohort had the p.Tyr19* (Y19X) mutation (F14), resulting in a non-functional protein (19). Despite carrying the same mutation, siblings in this family exhibited different clinical symptoms (**Table 2**). The lack of adrenal insufficiency in the youngest sibling may be age-related, as the condition can develop later in life.

Another set of siblings (F4) carried the c.885G>A (p.Trp295* or W295X) mutation, first described in 2001 (20). This mutation creates a premature stop codon, causing mRNA degradation (35). Both male siblings from this family developed hypertensive crisis and early neurological and autonomic dysfunction, while the female sibling with the same mutation had preserved neurological function.

Six patients from four families (Patients 12, 16, 17, 18, 30, 31) were identified with a novel c.852delG mutation. This mutation, a deletion of one base pair, leads to a predicted frameshift and a premature stop codon at the protein level (p.Trp284Cysfs*7). All truncating mutations in ALADIN up to amino acid 478 impair the normal function of the protein and, in the homozygous form, lead to triple-A syndrome (7). Interestingly, despite sharing the same mutations, these families (F8, F12, F13) who are from the same tribe and geographical area showed all three cardinal symptoms of triple A syndrome and no neurological involvement as of date. While another family (F20) with two affected siblings presented with full picture of triple A syndrome, including early neurological involvement, learning difficulties and thick nasal discharge, a previously unreported symptom.

Another novel mutation, c.394_399+2delCTGTCTGT, was identified in three male patients from two families (Patients 22, 23, 24) located at the border of exon 4 and intron 4 of the *AAAS* gene. This mutation affects the splicing of exon 4 and results on RNA-level in skipping of complete exon 4. On protein level skipping of exon 4 leads to an amino acid change of valine at position 103 to alanine and a frameshift with a premature stop codon eight amino acids later (p.Val103Alafs8*). Those patients from unrelated families and the same tribal ethnicity and geographical area had a similar set of symptoms, including alacrima, adrenal insufficiency, and neurological dysfunction, and no achalasia, possibly due to a younger age at presentation. One was also presented with excessive nasal discharge. The similarity in clinical symptoms among patients with the same novel mutation from the same tribe and geographical region suggests a possible role of genetic background in disease manifestation. However, this is contradicted by the significant clinical heterogeneity in symptoms observed among siblings from the same family, despite sharing the same mutation and tribal ancestry.

Triple A syndrome is a rare condition, with limited data from Sub-Saharan Africa and Arab countries. This study presents the largest comprehensive cohort of triple A patients from these countries. It highlights a wide range of clinical symptoms, including newly reported features and identifies a greater number of genetic mutations than previous studies from the region. In a Tunisian study, 26 patients were investigated, with 25 carrying the common Arabic founder mutation c.1331+1G>A, and one patient having the R286X mutation (16). A 2024 study of 10 Moroccan patients from seven families by Belmokhtar et al. found that all were homozygous for the c.1331+1G>A mutation (36). Other non-recurrent mutations such as c.1024C>T, c.1292-1294delTTCinsA, and c.1304delA have been found in North African patients (13, 37, 38).

In conclusion, this study is the first to provide both clinical and genetic insights into a large cohort of Sudanese patients with triple A syndrome. It sheds light on genetic mutations specific to Sub-Saharan Africa and the Arab population, where only a few reports have previously been reported from the region. This could enhance recognition and representation in similar ethnic and geographical populations where the condition is likely underdiagnosed due to limited awareness and diagnostic resources. Our findings also highlight a significant interfamilial and intrafamilial clinical variability, even among patients with the same mutation, suggesting that unlinked genetic and environmental factors may modulate the expression of the mutant genotype. This research fills a critical gap in understanding the diagnosis and genetic spectrum of this rare condition in a country with a high rate of consanguinity. Early diagnosis and treatment could potentially prevent mortality and significantly improve the quality of life for affected individuals.

## Data Availability

The original data presented in the study are included in the article. Further inquiries can be directed to the corresponding authors.
